# Potentially inappropriate medication use in the elderly in Montenegro

**DOI:** 10.1186/2050-6511-13-S1-A35

**Published:** 2012-09-17

**Authors:** Marta Rolevski, Berina Kučević, Gordana Boljević, Snežana Mugoša, Momir Mikov

**Affiliations:** 1 Faculty of Pharmacy, University of Montenegro, 81000 Podgorica, Montenegro; 2 Department of Pharmacology, Toxicology and Clinical Pharmacology, Faculty of Medicine, University of Novi Sad, 21000 Novi Sad, Serbia

## Background

Potentially inappropriate medications (PIMs) continue to be prescribed and used as first-line treatment for the most vulnerable of older adults, despite evidence of poor outcomes from the use of PIMs in older adults. PIM use is an important and preventable safety concern in the care of elderly patients and has been associated with adverse drug reactions, hospitalization and mortality. The aim of this study was to estimate the prevalence of PIM use among the elderly in Montenegro in 2012.

## Methods

The data about prescribed medications in the elderly were taken mostly from a data base of randomly selected general practitioners from various cities of Montenegro. Other sources of information were data bases of nursing homes. The study included information on 108 patients. PIMs were identified using the latest Beers criteria published in 2012. Results of our study were compared with similar researches that had been conducted in countries with a developed pharmacotherapeutic practice.

## Results

The study showed that 41.7% of Montenegrin patients received at least one PIM. Moreover, 31.1% of them used two or more PIMs. The most frequent PIMs, independent of diagnosis or medical condition, were NSAIDs (18.5%), followed by benzodiazepines (15.7%), digoxin (9.3%) and metoclopramide (3.7%). Potentially serious drug-drug interactions, according to the Beers criteria, were detected in 1% of all patients included in the study. Compared to this condition, for example, a study conducted in Sweden in 2005 [1] showed that the prevalence for PIMs was 17%; for anticholinergic drugs 6%, long-acting benzodiazepines 5%, psychotropic drugs 5% and potentially serious drug-drug interactions 4%. Logistic regression revealed no direct correlation between the total number of medications taken and the likelihood of receiving an inappropriate drug in Montenegrin patients.

## Conclusions

Comparing the results of our study with results obtained in countries with a developed pharmacotherapeutic practice, it was shown that the use of PIMs in the elderly in Montenegro in 2012 was significantly higher. Based on the results of our study, the use of drugs among elderly in Montenegro, in most cases, doesn’t conform to the principles of rational pharmacotherapy practice. This study emphasizes the need for continued provider education to inform prescribers of the potential risks of using certain medications in the elderly and to improve prescribing practices.
